# *From Kitchen to Clinic*: Study protocol for a family-centered, food-as-medicine approach using medically tailored meals and caregiver coaching in pediatric oncology care

**DOI:** 10.1016/j.conctc.2026.101681

**Published:** 2026-08-29

**Authors:** Brandy-Joe Milliron, Haley Schlechter, Cass Muir, Cynthia Moss, Jule Anne Henstenburg, Kylee Rappaport, Faustina Boakye, Jonathan M. Deutsch, Tracey Jubelirer

**Affiliations:** a Department of Health Sciences, College of Nursing and Health Professions, Drexel University, Philadelphia, PA, USA; b Children's Hospital of Philadelphia, King of Prussia, PA, USA; c The Center for Childhood Cancer Research, Supportive Care in Oncology Research (SCORe) Program, Children's Hospital of Philadelphia, Philadelphia, PA, USA; d MANNA Institute, Metropolitan Area Neighborhood Nutrition Alliance, Philadelphia, PA, USA; e Department of Pediatrics, University of Pennsylvania, Perelman School of Medicine, Philadelphia, PA, USA; f Division of Oncology, Children's Hospital of Philadelphia, Philadelphia, PA, USA

**Keywords:** Pediatric oncology, Medically tailored meals, Food as medicine, Caregiving, Nutrition, Survivorship, Feasibility

## Abstract

**Background:**

Children undergoing cancer treatment are at high risk for malnutrition, poor diet quality, and early cardiometabolic risk due to treatment-related side effects, disrupted appetite, and household-level barriers to healthy eating. Caregivers play a central role in nutritional support during treatment but often experience substantial burden, time constraints, and nutrition insecurity. Food-as-medicine strategies, including medically tailored meals (MTMs), have shown promise in adult populations but remain underexplored in pediatric oncology, particularly during active treatment and within family-centered models.

**Objective:**

The primary objective of this study is to evaluate the feasibility, acceptability, and adherence of *From Kitchen to Clinic*, a 12-week, family-centered food-as-medicine intervention that integrates MTM delivery with caregiver coaching for caregivers of children undergoing cancer treatment. Secondary objectives include collecting exploratory data on patient dietary intake, weight and lean body mass trajectories, treatment tolerance, household food and nutrition security, and caregiver burden and distress to inform a future definitive trial.

**Methods:**

This prospective, single-arm feasibility study will enroll 60 caregiver–child dyads (N = 120) recruited from three outpatient pediatric oncology clinics at a large children's hospital. The 12-week intervention includes an intensive phase (weeks 1–8) with weekly MTM delivery and caregiver coaching, followed by a tapered phase (weeks 9–12) with reduced meal frequency and bi-weekly coaching. Feasibility outcomes include recruitment, retention, assessment completion, intervention adherence, and acceptability, evaluated against predefined progression criteria. Exploratory outcomes will be assessed at baseline and at 3, 6, and 12 months post-intervention using validated dietary, anthropometric, clinical, and caregiver-reported measures.

**Trial registration:**

NCT07454902.

## Highlights

• Family-centered food-as-medicine intervention during pediatric cancer treatment.
• Combines medically tailored meals with caregiver coaching.
• Evaluates feasibility, acceptability, and adherence using predefined criteria.
• Addresses nutrition security and caregiver burden during active treatment.

## 1 Introduction

Children undergoing cancer treatment are at high risk for malnutrition, including both undernutrition (e.g., inadequate nutrient intake, weight loss, growth faltering) and overnutrition (e.g., excess weight gain, obesity) [[1], [2], [3]]. These outcomes are driven by the side effects of chemotherapy, corticosteroids, and radiation, which disrupt appetite, metabolism, gastrointestinal function, and physical activity [1,4]. As survival rates for childhood cancers continue to improve, weight-related late effects, including obesity, insulin resistance, dyslipidemia, and other cardiometabolic abnormalities, have emerged as prevalent and clinically significant contributors to long-term morbidity, diminished quality of life, and premature cardiovascular disease among survivors [[5], [6], [7], [8], [9]].

Overweight and obesity frequently develop early during treatment, particularly among children with acute lymphoblastic leukemia, and often persist into survivorship [4,10,11]. Cardiovascular disease is now a leading non-cancer cause of morbidity and mortality among childhood cancer survivors, with risk amplified by both treatment-related cardiotoxicity and modifiable lifestyle factors such as diet quality, excess adiposity, and physical inactivity [9,12,13]. Importantly, evidence suggests that cardiometabolic risk trajectories may be established during active treatment, underscoring early therapy as a critical window for preventive intervention rather than deferring lifestyle support to survivorship alone [4,10].

Caregivers, who are often the primary source of nutritional support during treatment, face substantial stress and competing demands related to intensive treatment schedules, symptom management, and financial strain, which can limit their capacity to provide nutritionally adequate meals [3,14]. These challenges contribute to household-level nutrition insecurity and can further compromise dietary quality and treatment adherence [4,8]. Treatment-related symptoms, including dysgeusia, nausea, fatigue, mucositis, and appetite changes, frequently lead children to prefer energy-dense, highly processed foods, contributing to poor diet quality during treatment and the persistence of unhealthy dietary patterns into survivorship [1,3,4]. Compared with pre-diagnosis dietary behaviors, children undergoing cancer treatment often consume inadequate fruits and vegetables and excess ultra-processed foods, patterns that may compound long-term cardiometabolic risk [1,8,15].

Diet quality is a modifiable risk factor for cardiometabolic health among childhood cancer survivors. Observational evidence suggests that greater adherence to healthy dietary patterns is associated with more favorable cardiovascular risk profiles in survivors, particularly those already exposed to cardiotoxic therapies [8]. Early lifestyle interventions initiated during cancer treatment have been shown to be feasible and safe, with emerging evidence of modest improvements in dietary intake and cardiometabolic markers; however, sustained, scalable, and family-centered nutrition interventions during active treatment remain limited [4,10].

Food-as-medicine interventions, particularly medically tailored meals (MTMs), are gaining momentum as promising strategies to address both medical and social needs through food-based healthcare [3,16]. MTMs provide convenient, nutritionally appropriate meals aligned with treatment goals while directly addressing barriers common during pediatric cancer treatment, including food access constraints, caregiver time scarcity, treatment-related fatigue, and symptom-driven food preferences. [14,16] In adult populations with nutrition-sensitive chronic conditions, MTMs have been shown to improve diet quality, reduce food insecurity, and, in some studies, lower disease-specific hospitalizations and short-term mortality, although effects on overall healthcare utilization have been mixed [[17], [18], [19]]. Although evidence from oncology populations remains limited, emerging qualitative and mixed-methods evidence suggests that MTMs, particularly when paired with nutrition counseling, may support patient engagement, reduce food-related stress, and facilitate active coping and behavior change during active cancer treatment; however, such studies remain few and have largely been limited to adult populations [16,20]. Despite strong theoretical alignment with the needs of pediatric oncology populations, MTMs and other food-as-medicine approaches remain underexplored in children undergoing cancer treatment, particularly as part of early, family-centered preventive strategies.

### 1.1 Objectives

Taken together, these findings highlight a critical gap in pediatric oncology care: the lack of pragmatic, family-centered nutrition interventions initiated during active treatment that address both treatment-related symptoms and household-level barriers to healthy eating. To address this gap, we developed *From Kitchen to Clinic*, a family-centered, food-as-medicine intervention that integrates MTMs with caregiver coaching. The primary objective of this feasibility study is to provide evidence for the feasibility, acceptability, and adherence of a 12-week food-as-medicine intervention, combining MTM delivery with caregiver coaching. The secondary objective is to collect exploratory outcome data on patient dietary intake, patient weight outcomes, and treatment tolerance; household food and nutrition security; and caregiver burden and distress. These data will be used to estimate variability, assess measurement procedures, and identify potential signals of intervention effect to inform the selection of outcome measures and sample size calculations for a future trial.

## 2 Methods

### 2.1 Study design

**Fig. 1:**
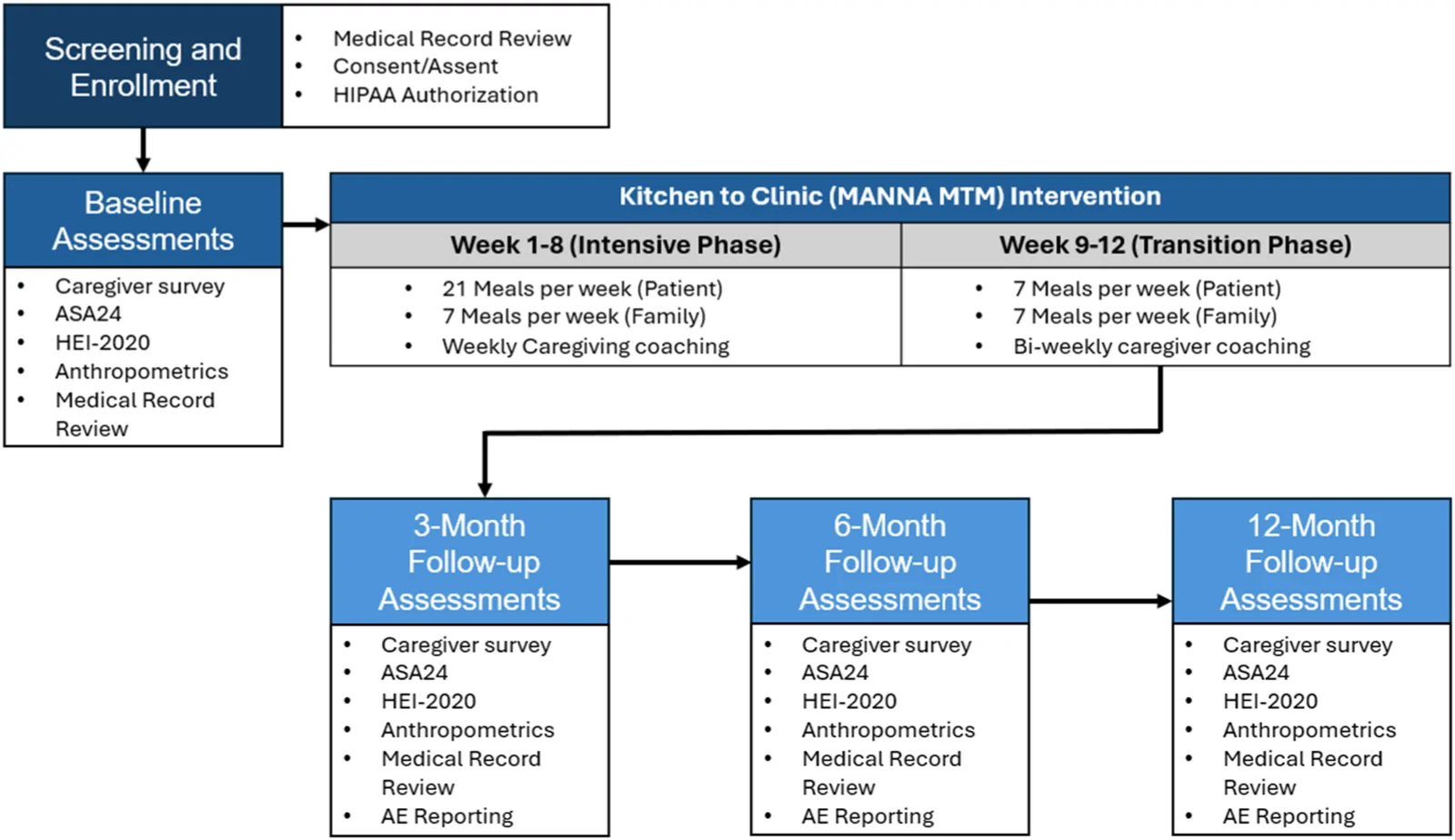
Study schema.

This is a prospective single-arm feasibility study and will include children on active cancer treatment and caregivers (n = 60 dyads). Caregiver-child dyads, where caregiver is defined as a parent or legal guardian of the child receiving treatment for cancer, will be recruited from three outpatient oncology clinics at the Children's Hospital of Philadelphia (CHOP). This study was approved by the Institutional Review Board at CHOP (IRB 26-024340) and registered at ClinicalTrials.gov (NCT07454902). See Fig. 1 for the study schema.

### 2.2 Study population and sample size

In this study, caregiver-child dyads in which the child is actively receiving cancer treatment will be invited to participate in a food-as-medicine intervention. Consistent with the aims of early-phase research, this feasibility study is not powered to detect statistically significant intervention effects; rather, its primary purpose is to assess feasibility and acceptability and to generate data that will inform the design of a subsequent, fully powered trial. A total of 60 caregiver-child dyads (N = 120; 60 children and 60 caregivers) will be enrolled using convenience sampling.

This sample size is consistent with methodological recommendations for pilot and feasibility studies, which suggest that samples of approximately 50-60 participants are common and adequate for evaluating key feasibility outcomes such as recruitment, retention, intervention adherence, and acceptability, particularly when multiple feasibility indicators are of interest [21,22]. Prior reviews of feasibility studies report a median total sample size of approximately 60 participants and note that samples in this range are sufficient to characterize feasibility parameters with reasonable precision while minimizing participant burden and resource use [22]. The proposed sample will allow estimation of feasibility metrics and associated confidence intervals to guide progression criteria and inform planning of a future randomized trial.

### 2.3 Eligibility criteria

Patients must be anticipated to receive treatment for their cancer diagnosis for at least 12 weeks. Children must be between five and 21 years of age at the time of enrollment, be able to speak English, and be approved to participate from the study's clinical Principal Investigator (PI). Patients must be anticipated to reside in the same household as their designated caregiver for the duration of the study. Patients will be excluded if they have swallowing or digestive disorders, food allergies (e.g., celiac disease, lactose intolerance, nut allergy), or specific food preferences that cannot be accommodated by the meal provider; have a diagnosis managed by neuro-oncology or a diagnosis of Acute Myeloid Leukemia (AML); or are undergoing bone marrow transplantation.

Eligible caregivers must be a parent or legal guardian of a child who meets all patient inclusion and exclusion criteria. Caregivers must be at least 18 years of age, able to read and speak English, have access to an internet-enabled device (e.g., smartphone, tablet, laptop, or desktop computer), and have the capacity to provide informed consent. Caregivers must also be anticipated to reside in the same household as the participating child for the duration of the study, and must live within the Metropolitan Area Neighborhood Nutrition Alliance (MANNA) meal delivery radius. Individuals who do not meet all eligibility criteria will not be enrolled.

### 2.4 Consent and enrollment

Potentially eligible caregiver–child dyads will be identified in collaboration with the pediatric oncology care team and screened for eligibility by study staff. Families meeting preliminary eligibility criteria will be approached during routine clinical encounters or contacted by phone or secure electronic communication. Study staff will provide a detailed explanation of the study purpose, procedures, risks, and benefits and allow sufficient time for questions prior to enrollment.

Written informed consent will be obtained from caregivers for both caregiver and child participation. For pediatric participants younger than 18 years of age, parental permission will be obtained from the caregiver, and written informed assent will be obtained from children aged 7 years and older, in accordance with institutional policies. Written HIPAA authorization will be required for child participation, as study staff will extract selected demographic and clinical information from the electronic medical record, including age, sex, race and ethnicity, diagnosis, treatment details, date of diagnosis, relapse status, cancer type and treatment at first diagnosis if relapse has occurred, and zip code of residence.

Enrollment will occur only after confirmation of eligibility and receipt of all required consent, assent, and authorization documents. Participation is voluntary, and caregivers and patients may withdraw at any time without impact on clinical care. All consent and enrollment procedures will be conducted in accordance with institutional review board approval.

### 2.5 Intervention

Meals for this study will be provided by the MANNA, a Philadelphia-based nonprofit organization that prepares and delivers MTMs for individuals with serious illnesses, including cancer. MANNA's MTM program aligns with evidence-based nutrition guidelines while accommodating medical diagnoses and select dietary preferences. Meals are developed collaboratively by Registered Dietitian Nutritionists (RDNs) and professional chefs and prepared onsite in MANNA's commercial kitchen using fresh, nutrient-dense ingredients. For this study, MANNA will provide kid-friendly, ready-to-eat/heat meals that are aligned with oncology nutrition guidelines and designed to support adequate energy and protein intake. Dietary restrictions that cannot be accommodated by MANNA are assessed during enrollment to ensure appropriateness.

Once participants are enrolled in the study, their information will be transmitted to MANNA via a standardized referral form completed by the study team. This referral is sent to MANNA's Client Services Department, which then assumes responsibility for onboarding families into the MTM program. A MANNA representative contacts the caregiver directly to initiate services and schedule meal delivery. During this intake call, caregivers are asked about dietary restrictions, food preferences, and medical considerations; accommodations are made when feasible and consistent with MANNA's available diet modifications. Dietary preferences and restrictions may be updated throughout the study period as needs change. Families select a preferred weekly delivery day, and adjustments to upcoming deliveries can be accommodated if requested at least two days prior to the scheduled delivery. At the start of service, families receive a MANNA-provided booklet outlining safe storage and reheating instructions, as well as contact information for questions, concerns, or troubleshooting. Caregivers may request changes to meal frequency (e.g., temporary reduction in meals) at any time, and all change requests can be communicated either directly to MANNA or through study staff, who will coordinate with MANNA to ensure continuity of services and alignment with study procedures.

*From Kitchen to Clinic* is a family-centered, food-as-medicine intervention combining MTMs and caregiver coaching with the goal of reducing treatment-related barriers to healthy eating and supporting families during a critical early period following diagnosis. The intervention is delivered over a 12-week period and consists of two phases: an intensive phase (weeks 1–8) followed by a tapered phase (weeks 9–12). During the intensive phase, participating families will receive a higher frequency of medically tailored meals to provide consistent nutritional support during the early, most treatment-intensive period following diagnosis. In this phase, families will receive a weekly delivery of 21 pre-cooked meals (7 breakfasts, 7 lunches, 7 dinners, healthy desserts, and fruit) for the pediatric patient, and 7 meals per week provided for the family to use as they desire (families are also able to opt-out of the 7 family meals, and those data will be included in our feasibility and acceptability analyses). Caregivers will also receive weekly one-on-one coaching sessions (remote or in-person, depending on caregiver preference) with a RDN. Sessions will focus on building caregiver skills, knowledge, and confidence in providing nutrition support, managing treatment-related challenges, and incorporating MTMs into family routines.

During the tapered phase (weeks 9–12), the frequency of meal delivery and coach contact will be reduced to support transition toward greater caregiver autonomy and sustainability of dietary behaviors beyond the intervention period. During this phase, families will receive a weekly delivery of 7 meals (1 meal per day) for the pediatric patient, while family meal support remains consistent (7 per week). Caregiver coaching will transition to bi-weekly sessions, providing continued support while encouraging autonomy. This stepped-down approach is intended to balance ongoing support with skill-building and prepare families for independent meal planning as treatment routines stabilize.

### 2.6 Data collection schedule

Data will be collected at four primary time points: baseline (prior to or at initiation of the intervention), and post-intervention follow-up at 3, 6, and 12 months. Baseline assessments will occur after informed consent and assent are obtained and before delivery of intervention components. Post-intervention assessments will be conducted immediately following completion of the intervention period, and follow-up assessments will be conducted to evaluate short-term maintenance and feasibility of continued data collection. Feasibility metrics, including recruitment, retention, adherence to intervention components, assessment completion, and intervention engagement, will be tracked continuously throughout the study. Data collection at baseline, 3, 6, and 12 months will include patient dietary intake, weight outcomes, and treatment tolerance; household food and nutrition security; and caregiver burden and distress. The timing and burden of assessments will be monitored to inform optimization of the protocol for future trials. See Table 1 for the schedule of procedures.

**Table 1:** Schedule of study procedures.

Study Activity	aScreening/Enrollment	Intervention Period			Follow-up Period		
		aBaseline (+14 days)	aWeek 1-8 (Intensive)	aWeek 9-12 (Transition	a3 Month (+28 days)	a6 Month (±28 days)	a12 Month (±28 days)
Eligibility screening	X						
Informed consent/assent	X						
Medical record review	X	X	X	X	X	X	X
Anthropometrics: height, weight, BMI z-score		X			X	X	X
Mid-upper arm circumference (MUAC)		X			X	X	X
Medically Tailored Meals		X	$X^{1}$	$X^{1}$			
Caregiver Coaching Session			$X^{2}$	$X^{2}$			
Caregiver Personal Characteristics survey		X					
Challenges and Goals survey		X					
Resources and Preferences for Supportive Care survey		X					
Caregiving Experience survey		X					
Meals and Meal-time Behaviors survey		X					
Food Security survey		X			X	X	X
Healthfulness Choices survey		X			X	X	X
Nutrition Security survey		X			X	X	X
PROMIS Emotional Distress – Anxiety survey		X			X	X	X
PROMIS Satisfaction with Social Roles/Activities survey		X			X	X	X
Intervention Acceptability survey					X		
Meal Adherence survey			$X^{3}$	$X^{3}$			
Adverse Event Reporting			X	X	X	X	X
Child Dietary Intake (ASA24)		$X^{4}$			$X^{4}$	$X^{4}$	$X^{4}$
Healthy Eating Index (HEI-2020)		$X^{5}$			$X^{5}$	$X^{5}$	$X^{5}$

1: MTM- 21 meals for patient, 7 meals for family per week; weekly caregiver one-on-one coaching session.

2: MTM- 7 meals for patient, 7 meals for family per week; bi-weekly caregiver one-on-one coaching session.

3: Caregiver will complete survey weekly during the intervention period.

4: Via caregiver proxy; 3 recalls: 2 weekday, 1 weekend.

5: Scored form ASA24.

a The study activities do not need to occur on the same day based on visit type.

### 2.7 Outcomes

The feasibility, acceptability, and intervention adherence outcomes described below and outlined in Table 1, Table 2 are the primary outcome measures being assessed in this feasibility study. To characterize the sample, self-report information will be collected regarding race, ethnicity, sex, gender, income, education, child diagnostic and treatment information, and caregiving characteristics. Clinical and demographic information will be abstracted from participant charts by the study team. Information that is not collected as part of routine care will be obtained directly from the caregiver.

**Table 2:** Feasibility and acceptability progression criteria.

Outcome	Measure	Criteria
Recruitment feasibility	Participants enrolled per month of recruitment	≥3 dyads/month
Retention feasibility	Percent of participants providing assessment data at 12 weeks	≥75%
Assessment feasibility	The proportion of retained participants who provide valid data for each assessment task	≥85%
Intervention acceptability	Intervention acceptability survey Domains: Items grouped into three subscales: Meals Acceptability (items 4–8) Coaching Acceptability (items 9–12) Overall Program Acceptability & Integration (items 1–3, 13–15) *See Table 3 for details	Median score ≥4
Intervention adherence (Coaching and MTM adherence)	Caregiver coaching adherence (dose received): Attendance rate	≥75% of coaching sessions completed
	Child and family meals consumed will be assessed using three complementary sources: 1. Weekly 3-item caregiver survey Categorical (weeks 1-8) and numeric (weeks 9-12) responses converted to weekly MTM uptake (%) and verified through coaching and dietary recall. 2. Coaching call confirmation 3. Dietary recall cross-check	≥70% of provided child meals consumed per week during weeks 1–8, and ≥60% during the taper (weeks 9–12)
	The previous three sources will be used to calculate and confirm: • % of provided child meals consumed • % of family meals consumed	≥60% of provided family meals used per week
	A composite adherence score will be calculated (0-100 index each week; averaged across weeks)	Composite adherence score: High: ≥75 Moderate: 50–74 Low: <50
	Weekly Adherence Index = 0.50 × Child MTM uptake (%) + 0.20 × Family MTM uptake (%) + 0.30 × Coaching session completion (%)	
Progression criteria	Feasibility will be determined by whether the intervention meets at least 4 out of 5 of the defined feasibility progression criteria. For intervention adherence, criteria for adherence will be considered a composite score of 75 or greater. Meeting these progression criteria will support the decision to proceed to a future definitive trial.	

#### 2.7.1 Primary outcomes

Consistent with Mellor et al.’s recommendations for feasibility trials, we have defined specific progression criteria to determine whether the study procedures are practicable and whether to proceed to a larger trial [23].

• **Recruitment feasibility (≥3 dyads/month):** This benchmark is informed by recruitment rates achieved in prior studies at the participating clinical sites and reflects both available research staff capacity and the anticipated burden on caregivers of children undergoing cancer treatment. Recruitment rates below this threshold may suggest limited reach, acceptability, or scalability of the intervention in its current form.
• **Retention (≥75%) and assessment completion (≥85%) feasibility:** At least 75% of enrolled participants are expected to complete post-intervention assessments at 8 weeks, and at least 85% of participants retained at that time point should provide valid data for each assessment measure. These thresholds are consistent with feasibility and pilot study guidance indicating that retention rates of approximately 70-80% are generally required to support progression to a larger trial, particularly in vulnerable populations [21,24,25]. High assessment completion among retained participants will further indicate that study procedures are acceptable and not unduly burdensome.
• **Intervention Adherence:** Adherence to the intervention will be evaluated as part of feasibility outcomes. For caregiver coaching, adherence will be defined as the proportion of scheduled sessions completed (target ≥75%). This threshold aligns with criteria used in similar behavioral interventions, where attending at least 75% of sessions is associated with increased retention of nutrition knowledge and meal behavior change. Weekly adherence will be derived from caregiver-completed meal adherence surveys, including reasons for missed meals. During weeks 1-8, categorical responses will be converted to estimated meal counts using pre-specified midpoint scoring rules. During weeks 9-12, numeric responses will be scored directly. These values will be converted to weekly MTM uptake percentages. Coaches will corroborate uptake during calls, and dietary recalls (ASA24) will include a tag to identify days when MANNA meals were consumed. MTM adherence will be summarized as the proportion of provided meals consumed (target ≥70% uptake during weeks 1–8 and ≥60% during taper weeks 9–12). These adherence metrics will be analyzed descriptively and used to interpret feasibility, acceptability, and exploratory clinical outcomes.
• **Acceptability**: Domain scores (Meals, Coaching, and Overall/Integration) will be calculated as the mean of relevant items, and an overall acceptability index will be computed as the mean of all items. In alignment with feasibility benchmarks, the median score of the global item (“The overall Food-as-Medicine program was acceptable to me and my family”) will also be reported, with a threshold of ≥4 indicating strong acceptability. Additional questions will be asked, including if and how the meals affected family's daily routine, favorite and least favorite meals, and the most common reasons for child refusal of meals, among others. Both mean (SD) and median (IQR) values will be presented to characterize response distributions, and open-ended questions will provide qualitative insights to contextualize survey findings (Table 3).

**Table 3:** Intervention acceptability survey domains and scoring.

Domain	Items	Sample Item	Scoring Approach
Overall Intervention	3	“The overall Food-as-Medicine program was acceptable to me and my family.”	Mean of items 1–3; also report median of the global item (Item 1) for feasibility benchmark (≥4 = acceptable).
Medically Tailored Meals (MTMs)	5	“The meals helped reduce the time and stress associated with meal planning and preparation.”	Mean of items 4–8 (1 = Strongly Disagree to 5 = Strongly Agree).
Caregiver Coaching	4	“The coaching sessions were useful for managing my child's nutrition during treatment.”	Mean of items 9–12.
Integration & Sustainability	3	“The combination of meals and coaching worked well together.”	Mean of items 13–15.
Burden & Accessibility	1	“Participation in the study did not feel overly burdensome to my schedule.”	Single item (Item 16), reported descriptively.
Total Acceptability Index	All 16	N/A	Mean of all completed items (≥75% completion required). Higher scores = greater acceptability.

Notes:-Each item uses a 5-point Likert scale: 1 = Strongly Disagree, 2 = Disagree, 3 = Neutral, 4 = Agree, 5 = Strongly Agree.- For all domains and the total index, both mean (SD) and median (IQR) will be reported.- Qualitative open-ended responses will supplement quantitative scoring to contextualize acceptability findings.
• **Progression Criteria**: Feasibility will be determined by whether the intervention meets at least 4 out of 5 of the defined feasibility criteria. Meeting these criteria will support the decision to proceed to a future definitive trial.

#### 2.7.2 Secondary outcomes

Exploratory outcome data will be collected at baseline and post-intervention follow-up at 3, 6, and 12 months. These data will include patient dietary intake, patient weight and lean body mass trajectories, patient treatment tolerance, household food and nutrition security, and caregiver burden and distress. A medical record review will be conducted to collect data related to cancer treatment tolerance, including dose/dose modifications, treatment delays, lengths of hospitalizations, and toxicity. Anthropometric data will be collected at each assessment visit including height, weight, BMI z-score (calculated), and mid upper arm circumference. Questionnaires will be sent to the caregivers via REDCap if they are unable to complete them in clinic. Caregiver reported data collected at baseline only will include personal characteristics (medical history, demographic information, time spent caregiving), participation in community and federal food and nutrition assistance programs, meal and meal-time behaviors, and preferences for supportive care. Additionally, caregivers will be asked to share the reasons for joining the study and nutrition challenges they are experiencing.

Food security will be assessed using the two-item *Hunger Vital Signs* screener, which asks caregivers about food availability at home. Responses are reported anchored by the words “Often true”, “Sometimes true”, and “Never true” [26,27]. Household nutrition security and food choice healthfulness will be assessed using adapted survey instruments developed by Calloway and colleagues [28]. The *Nutrition Security* module (4 items) evaluates whether families have consistent access to foods that meet their nutritional needs, while the *Healthfulness of Choice* module (3 items) assesses the quality and healthfulness of food options available and selected within the household. Each instrument contains short questions designed to capture constructs beyond traditional food insecurity, emphasizing nutritional adequacy and choice characteristics. These tools have been shown to be feasible, reliable, and valid for use in community-based studies, and they provide an expanded perspective on how households experience and manage nutritional insecurity. In this study, items and response options were unchanged; only the recall period was shorted to ‘past 3 months.’

Caregiver burden and distress will be assessed using standardized Patient-Reported Outcomes Measurement Information System (PROMIS) instruments. Specifically, caregivers will complete the PROMIS *Satisfaction with Social Roles and Activities* short form and the PROMIS *Emotional Distress - Anxiety* short form. The *Satisfaction with Social Roles and Activities* instrument measures the extent to which individuals are content with their ability to perform usual roles in work, family, and social life, providing insight into the functional impact of caregiving demands. The *Emotional Distress* instrument evaluates symptoms of anxiety and related emotional strain. PROMIS measures have been rigorously validated across diverse populations and use T-scores standardized to the U.S. general population (mean = 50, SD = 10), enabling comparability across studies.

*Patient dietary intake* will be assessed using the Automated Self-Administered 24-h Dietary Recall (ASA24), a free, validated, computer-based tool developed by the National Cancer Institute that provides standardized reporting of total caloric intake, macronutrients, micronutrients, and dietary patterns [29]. Caregivers will complete one 24-h recall on behalf of their child (patient) at each of the assessment time points (baseline, 3, 6, and 12 months). For the first recall for each participant, a trained research staff member will assist participants and will enter data into the ASA24 system on participants’ behalf. After the first recall, caregivers will be encouraged to complete subsequent recalls on their own. However, to reduce burden and support caregivers who require extra support, a research team member will be available to assist caregivers in all subsequent recalls.

### 2.8 Data analysis plan

Feasibility and acceptability will be evaluated as the primary outcomes and interpreted relative to predefined progression criteria (Table 2), consistent with published guidance for pilot and feasibility studies [22,23]. Criteria encompass recruitment, retention, assessment completion, intervention adherence, and acceptability and were selected based on prior literature, anticipated caregiver burden, and intervention logistics. Feasibility will be considered established if at least four of the five criteria are met, supporting progression to a future definitive trial.

Descriptive statistics will be selected based on variable type and distribution. Continuous variables will be summarized using means and standard deviations or medians with interquartile ranges, as appropriate, while categorical variables will be described using frequencies and percentages. Acceptability items measured on 5-point Likert scales (1 = strongly disagree to 5 = strongly agree) will be summarized using medians and interquartile ranges, with a median score ≥4.0 indicating acceptable or useful intervention components. Frequencies and percentages will also be presented to enhance interpretability. Distributional assumptions will be assessed through visual inspection.

Secondary endpoints will examine exploratory clinical and behavioral outcomes, including changes in patient dietary intake, weight and lean body mass trajectories, and treatment tolerability. Additional secondary outcomes will include changes in household food and nutrition security and caregiver burden and distress. Given the pilot nature of the study, these outcomes will be analyzed descriptively by comparing pre- and post-intervention measures to inform future hypothesis-driven trials.

### 2.9 Potential risks and benefits

This study poses no greater than minimal risk, with anticipated risks comparable to those encountered during routine clinical care. Potential risks include dissatisfaction with meal taste or variety; inadvertent exposure to food allergens or intolerances; transient gastrointestinal symptoms (e.g., bloating, constipation, diarrhea) related to dietary changes; emotional discomfort during nutrition- or caregiving-related discussions; and risks to privacy or confidentiality associated with collection of personal health information. Food preferences, allergies, and intolerances will be assessed at enrollment, and meals will be tailored within the constraints of the meal provider's standard offerings. Meals align with oncology nutrition guidelines, and any nutrition-related concerns or symptoms will be reviewed by the study dietitian. Coaching staff are trained in supportive communication, and participants experiencing emotional distress will be referred to institutional psychosocial support services. Risks related to privacy and confidentiality will be minimized through established data security and confidentiality procedures.

Participants may experience several potential benefits. Children may benefit from access to medically tailored meals designed to support adequate nutrient intake, weight stability, and management of treatment-related side effects. Caregivers may benefit from reduced burden related to meal planning and preparation, improved household nutrition security, and increased knowledge, skills, and confidence in supporting their child's nutrition through individualized coaching. The combined provision of meals and coaching may help reduce caregiver distress and support the development of sustainable, healthy eating practices beyond the intervention period. At a broader level, this study will contribute to understanding the feasibility and potential impact of food-as-medicine interventions in pediatric oncology and may inform future programs to support families during cancer treatment.

### 2.10 Safety reporting

Adverse events (AEs) that are unexpected and deemed at least possibly related to study participation will be documented and graded using the Common Terminology Criteria for Adverse Events (CTCAE v5.0). During coaching sessions, caregivers will be asked about concerns related to meals, treatment-related nutrition side effects, or other health events, and unresolved reportable AEs will be followed until resolution. Given the minimal-risk nature of this dietary intervention, unrelated or clearly incidental events will not be recorded. Serious adverse events (SAEs) resulting in death or deemed life-threatening will be reported to the IRB within 48 h of notification; all other related SAEs or unanticipated problems will be reported within 7 days. AE documentation will be maintained in REDCap and reviewed biannually, with summary reporting at continuing review. Early stopping is not anticipated; however, the study may be suspended or terminated in the event of unexpected related SAEs, poor recruitment or retention, or protocol deviations that compromise safety or data integrity. A formal Data and Safety Monitoring Board is not required for this minimal-risk pilot study.

## 3 Discussion

This protocol describes *From Kitchen to Clinic*, a prospective, single-arm feasibility study evaluating a 12-week, family-centered food-as-medicine intervention for caregivers of children undergoing cancer treatment. The intervention integrates MTM delivery with caregiver coaching, delivered during a critical period following diagnosis when treatment intensity, caregiver burden, and nutrition-related challenges are often greatest. The stepped intervention design - featuring an intensive phase followed by a tapered transition phase - was intentionally selected to balance early support with skill-building and sustainability, while minimizing participant burden.

Despite growing recognition of nutrition as a modifiable contributor to long-term survivorship outcomes, there remains a lack of pragmatic, family-centered nutrition interventions delivered during active pediatric oncology treatment. *From Kitchen to Clinic* addresses this gap by combining MTMs with caregiver coaching to reduce structural and psychosocial barriers to healthy eating. By intervening early, during active treatment rather than survivorship alone, this study aligns with emerging evidence suggesting that dietary patterns, weight trajectories, and cardiometabolic risk may be established during treatment and persist thereafter. This protocol contributes to the limited but growing literature on food-as-medicine approaches in oncology and represents one of the few studies to examine MTMs within a pediatric, family-centered model.

Several challenges are anticipated in the implementation of this study. Recruitment and retention may be affected by the competing demands faced by families during active cancer treatment, including frequent clinic visits, hospitalizations, and caregiving responsibilities. To mitigate these challenges, the study design emphasizes flexibility in scheduling, remote data collection options, and integration with existing clinical workflows. Participant burden related to repeated assessments and dietary recalls may also impact completion rates; therefore, feasibility outcomes related to assessment completion and participant feedback will be closely monitored. Intervention adherence may vary due to treatment-related symptoms, changes in appetite, or evolving family preferences. The inclusion of caregiver coaching and a tapered intervention phase is intended to support engagement while allowing adaptation to individual family circumstances. Findings related to adherence and acceptability will be critical for refining intervention dose, delivery, and support strategies in future trials.

A key strength of this study is its focus on scalability and real-world implementation. The intervention leverages an established community-based MTM provider and integrates RDN coaching in a manner that could be embedded within standard supportive oncology care. By systematically evaluating feasibility metrics and progression criteria, this study will generate actionable data to inform whether and how the intervention could be expanded to additional clinical sites or populations. If feasibility benchmarks are met, findings from this study will inform the design of a future randomized controlled trial to evaluate effectiveness. Additionally, the integration of household-level outcomes, caregiver-reported measures, and implementation-relevant data positions *From Kitchen to Clinic* to contribute to policy-relevant discussions around food-as-medicine models in pediatric oncology and broader supportive care contexts.

Several considerations will be important when designing subsequent evaluations of this intervention. The present study was intentionally designed as a single-arm feasibility study and therefore does not evaluate the feasibility or acceptability of a comparison condition. This is particularly relevant for a MTM intervention because receipt of the intervention is readily apparent and represents a substantial form of material support. In a future randomized trial, participants assigned to a non-meal condition may have different expectations or experience disappointment related to treatment assignment, which could influence engagement, retention, and patient-reported outcomes, particularly psychosocial measures [30,31].

Accordingly, future studies should carefully evaluate both the content of the comparison condition and the overall trial design. Potential comparators could include usual care, enhanced usual care, or an attention-control condition. Participant-preference designs may also warrant consideration given the tangible nature of the intervention [32]. For example, a partially randomized preference trial could allow participants with strong treatment preferences to select a condition while randomizing those without a strong preference, whereas a conventional randomized trial could prospectively assess treatment preference and examine whether preference modifies adherence, retention, or outcomes. Although preference-based approaches may reduce concerns related to disappointment and differential retention, they also introduce potential selection bias and greater complexity in estimating causal intervention effects. These design considerations should be explored as the intervention progresses from feasibility testing to larger-scale effectiveness evaluation.

In summary, *From Kitchen to Clinic* is a novel, family-centered feasibility study designed to address a critical gap in pediatric oncology care by integrating medically tailored meals with caregiver coaching during active treatment. By evaluating feasibility, acceptability, and adherence, and generating preliminary data on dietary, household, and caregiver outcomes, this study will provide essential groundwork for future trials and implementation efforts. Ultimately, this work aims to advance the role of food-as-medicine approaches in pediatric oncology and inform scalable strategies to support children and families during cancer treatment.

## CRediT authorship contribution statement

**Brandy-Joe Milliron:** Writing – review & editing, Writing – original draft, Supervision, Resources, Methodology, Investigation, Funding acquisition, Conceptualization. **Haley Schlechter:** Conceptualization, Investigation, Methodology, Writing – review & editing. **Cass Muir:** Methodology, Project administration, Supervision, Writing – review & editing. **Cynthia Moss:** Methodology, Project administration, Writing – review & editing. **Jule Anne Henstenburg:** Methodology, Writing – review & editing. **Kylee Rappaport:** Project administration, Writing – review & editing. **Faustina Boakye:** Methodology, Writing – review & editing. **Jonathan M. Deutsch:** Methodology, Resources, Writing – review & editing. **Tracey Jubelirer:** Conceptualization, Funding acquisition, Investigation, Methodology, Resources, Writing – review & editing.

## Ethics approval and clinical trial registration

The trial was granted full ethics approval by the Institutional Review Board at CHOP (IRB 26-024340). The trial is registered with ClinicalTrials.gov ID: NCT07454902.

## Funding

Funding support for this work was provided by the Metropolitan Area Neighborhood Nutrition Alliance (MANNA) and the Stein Bellet Foundation.

## Declaration of competing interest

The authors declare that they have no known competing financial interests or personal relationships that could have appeared to influence the work reported in this paper.

## Data Availability

No data was used for the research described in the article.
